# Modeling analysis revealed the distinct global transmission patterns of influenza A viruses and their influencing factors

**DOI:** 10.1111/1749-4877.12469

**Published:** 2020-08-06

**Authors:** Chaoyuan CHENG, Jing LI, Wenjun LIU, Lei XU, Zhibin ZHANG

**Affiliations:** ^1^ State Key Laboratory of Integrated Management on Pest Insects and Rodents in Agriculture Institute of Zoology Chinese Academy of Sciences Beijing China; ^2^ CAS Key Laboratory of Pathogenic Microbiology and Immunology Institute of Microbiology Chinese Academy of Sciences Beijing China; ^3^ Ministry of Education Key Laboratory for Earth System Modeling Department of Earth System Science Tsinghua University Beijing China; ^4^ University of Chinese Academy of Sciences Beijing China; ^5^ CAS Center for Excellence in Biotic Interactions University of Chinese Academy of Sciences Beijing China

**Keywords:** H1N1, H3N2, H5N1, H7N9, influenza, transmission pattern

## Abstract

Influenza A virus has caused huge damage to human health and poultry production worldwide, but its global transmission patterns and influencing factors remain unclear. Here, by using the Nearest Genetic Distance Approach with genetic sequences data, we reconstructed the global transmission patterns of 4 most common subtypes of influenza A virus (H1N1, H3N2, H5N1, and H7N9) and analyzed associations of transmission velocity of these influenza viruses with environmental factors. We found that the transmission patterns of influenza viruses and their associations with environmental factors were closely related to their host properties. H1N1 and H3N2, which are mainly held by humans, are transmitted between regions at high velocity and over long distances, which may be due to human transportation via airplane; while H5N1 and H7N9, which are mainly carried by animals, are transmitted locally at short distances and at low velocity, which may be facilitated by poultry transportation via railways or high ways. H1N1 and H3N2 spread faster in cold seasons, while H5N1 spread faster in both cold and warm seasons, and H7N9 spread faster in wet seasons. H1N1, H3N2, and H5N1 spread faster in places with both high and low human densities. Our study provided novel insights into the global transmission patterns, processes, and management strategies for influenza under accelerated global change.

## Graphical abstract

We developed a method named as Nearest Genetic Distance Approach (NGDA) by using available IAV genetic sequence data in the GenBank database to reconstruct the transmission routes and estimate the transmission velocity between two geographic sites. Using NGDA, we reconstructed the global transmission patterns for past decades of four most common subtypes (i.e. H1N1, H3N2, H5N1, H7N9) of IAVs and analyzed their associations of spread velocity of influenza viruses with environmental factors.

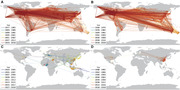

## INTRODUCTION

Influenza A viruses (IAVs) are enveloped negative‐sense single‐strand RNA viruses that can cause infectious respiratory disease to hosts (Krammer *et al*. 2018). IAVs are widely distributed in many hosts, mainly including birds, pigs, and humans (Kuiken *et al*. 2006; Taubenberger & Morens 2010), and have caused huge damage to human health and poultry production worldwide. It is estimated that the prevalence of influenza causes >3 million cases of severe illness to humans every year (WHO 2018). The IAV genome has a total of 8 RNA fragments encoding 14 structural proteins, and due to the high variation of hemagglutinin (HA) and neuraminidase (NA) fragments (2 major viral antigens), IAVs mutate and infect the host rapidly (Webster *et al*. 1992; Neumann *et al*. 2009). According to HA and NA fragments, the IAV is divided into many subtypes. H1N1 and H3N2 are 2 subtypes that currently circulate in humans (Krammer *et al*. 2018). H5N1 and H7N9 mainly infect chicken and birds (Krammer *et al*. 2018). IAVs can spread through contact, droplets, and airborne routes (Brankston *et al*. 2007), and its transmission is likely affected by humidity and temperature (Lowen *et al*. 2007).

Although the temporal dynamic of influenza viruses have been widely investigated (O'Regan *et al*. 2013; Yang *et al*. 2015; Huang *et al*. 2016; Wen *et al*. 2016; Cai *et al*. 2019), studies on the spatial‐temporal dynamic of influenza are still limited. Recently, probabilistic model‐based inference for phylogeography such as Bayesian Evolutionary Analysis Sampling Trees (BEAST) method is widely used to study the spatial‐temporal spread of pathogens like influenza between geographic locations (Lemey *et al*. 2009, 2010 ; Suchard *et al*. 2018). Several previous studies suggest that prevalence of influenza is associated with environmental factors. For example, in laboratory experiments, cold and dry conditions favor influenza virus transmission (Lowen *et al*. 2007). High temperature (30 °C) blocks aerosol but not contact transmission (Lowen *et al*. 2008), and high humidity constrains both transmission efficiency and influenza virus survival (Shaman & Kohn 2009). Influenza activity peaks in the cold‐dry season (Tamerius *et al*. 2013), and the transmission was negatively associated with the absolute humidity and positively associated with cumulative precipitation (Gomez‐Barroso *et al*. 2017). However, these studies are mainly constrained to transmission of influenza virus between hosts under laboratory conditions (Lipsitch & Viboud 2009; Neumann *et al*. 2009; Herfst *et al*. 2012; Neumann & Kawaoka 2015; Hill *et al*. 2016; Poon *et al*. 2016) or between locations at a small spatial‐temporal scale (Olsen *et al*. 2006; Viboud *et al*. 2006; Bedford *et al*. 2010; Gog *et al*. 2014; Huang *et al*. 2016; Pei & Shaman 2017; Pei *et al*. 2018). The transmission patterns and influencing factors of IAVs remain unclear at a global scale. There is an urgent need to reveal the global transmission patterns and the key factors affecting their transmission ability, so as to take effective measures of prevention and control.

Here, by following the molecular clock theory (King & Jukes 1967; Motoo Kimura 1968) and considering the fast but constant mutation rate of HA of IAV, we developed a method named as Nearest Genetic Distance Approach (NGDA) by using available IAV genetic sequence data in the GenBank database to reconstruct the transmission routes and estimate the transmission velocity between 2 geographic sites. Using NGDA, we reconstructed the global transmission patterns for past decades of 4 most common subtypes (i.e. H1N1, H3N2, H5N1, H7N9) of IAVs and analyzed their associations of spread velocity of influenza viruses with environmental factors.

## MATERIALS AND METHODS

### Sequences and environmental data

We collected the H1N1, H3N2, H5N1, and H7N9 influenza data from the GenBank database (https://www.ncbi.nlm.nih.gov/). The datasets used in this research included all infection events of 4 subtypes around the world. For each subtype, we extracted the sequences corresponding to the RNA strand encoding hemagglutinin (HA). The datasets included 76 032 records from the first case reported on May 11, 1918, to the latest case reported on October 30, 2018, and included the basic information (reported location and reported date) for each report. We assigned the sampling location with the latitude and longitude based on the administrative center coordinates using the “Geopy” package in Python 3.6.0 (https://www.python.org/).

We excluded samples without accurate sampling dates (daily resolution) or strain codes, as well as those with RNA sequence < 1600 bp (13.7% of the total sequences) in length. RNA sequences were aligned using MEGA7 (Kumar *et al*. 2016) with default parameters. We excluded country‐level samples with too large area (>3 00 000 km^2^, 7.6% of the total sequences). Finally, 6096 samples of H1N1 from 1279 locations, 5750 samples of H3N2 from 796 locations, 1046 samples of H5N1 from 531 locations, and 239 samples of H7N9 from 85 locations were used (Figs. S1 and S2, Supporting Information). The climatic data were collected from CRU (http://www.cru.uea.ac.uk/data), including the monthly average daily temperatures (MT) and monthly precipitation (MP). The yearly average temperature (YT) was calculated by averaging all MTs of the year. The annual precipitation (AP) of each year was calculated by summing all MPs of the year. The seasonal temperature (TempS) or precipitation (PrecS) of each month of a sample location was calculated by: MT‐YT or MP‐AP/12, representing the seasonal variation of these variables. The climate data is presented in netCDF4 format at a 0.5° × 0.5° resolution, covering the period of 1901–2017. Because the yearly data was limited, we focused on analyzing the effects of seasonal climate on transmission velocity of IAV. To remove the confounding effects of regional variation of climate, the seasonal climate was normalized.

Population density data of humans (person/km^2^) with a country‐level spatial resolution was obtained from the World Population Review (http://worldpopulationreview.com). We extracted data of relevant countries in 2018. Only population density was used to represent the impacts of human activities because gross domestic production and poultry farm density are highly correlated with population density.

### Reconstruction of transmission routes of IAVs

**Figure 1:**
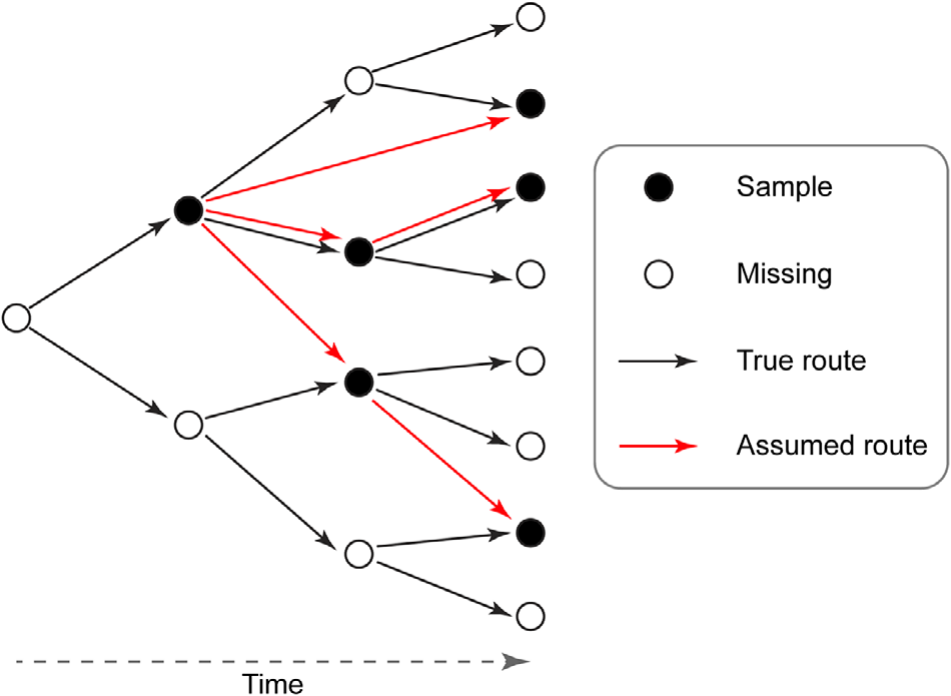
Illustration of Nearest Genetic Distance Approaches (NGDA) for reconstructing the transmission routes and calculating the transmission velocity of IAVs. Solid black and open circles represent the observed and missing samples of IVAs. The solid black arrows connecting the open circles represent the true transmission routes. The solid red arrows connecting the solid black circles represent the reconstructed transmission routes. The source sample collected previously to a focal sample was determined by the nearest genetic distance (for details, see the main text). Thus, the source sample of transmission represents either the direct ancestor or relative of the ancestor (proxy ancestor) of the focal sample. The mutation rate of virus was assumed to be constant in time.

We followed 3 rules by order to determine the transmission route from the source location to a focal invaded location: earlier sampling date, nearest genetic distance, and nearest geographic distance. We assumed that a previous sample with nearest genetic distance to a focal sample was most likely the source sample of transmission as the ancestor or a proxy ancestor to the focal sample (Fig. 1). If the genetic distance was equal, the sample with nearest geographic distance was assumed to be the source sample. We defined the method as the NGDA. It is notable that NGDA is different from the traditional phylogenetic tree analysis, which uses genetic data of extant animal samples. NGDA maps the spatial‐temporal transmission routes as well as lineages using genetic data of extinct virus or “fossil virus” because the sequenced virus was thought to be dead.

We aligned all the sequences in MEGA7, and then we compared each sample against all of the other samples (all‐versus‐all pairwise comparison) at each variant nucleotide position. The genetic distance between every pair of samples was defined as:

(1) $$d_{i,j}=m_{i,j}/n_{i,j}\;$$

Here, $d_{i,j}$ is the distance measured between samples *i* and *j*, $n_{i,j}$ is the total number of aligned nucleotide configurations (A, C, G, and T) between samples *i* and *j*, and $m_{i,j}$ is the total number of different nucleotides between samples *i* and *j*.

According to the molecular clock theory, the evolutionary rate at the molecular level is constant (King & Jukes 1967; Motoo Kimura 1968; Frost *et al*. 2018). We assumed that the mutation rate of the sequence of the IAV genome was stable over time, and this assumption was supported by previous studies (Lemey *et al*. 2009). The sampling time was the time when influenza virus survived because IAV cannot reproduce *in vitro*. During the study period, mutations can be accumulated at a high enough rate to calculate the differentiation of samples because the mutation rate of IAV is very fast (Drummond *et al*. 2003). The genetically nearest and previously collected sample was mostly likely the ancestor or relatives of the focal sample. We used the genetic distance (defined in Eqn 1), sampling date, and locations to determine the transmission routes.

For sample S_i_, we first found all samples with a sampling date earlier than S_i_, and then we calculated the genetic distance between S_i_ and these samples. The sample with the nearest genetic distance was assumed to be the transmission source of sample S_i_ (if not unique, the sample with the nearest geographical distance from these samples was the source of S_i_). The source sites of all of the samples were determined in such a way. Then, the global spread routes were ultimately reconstructed.

### Transmission velocity

After the transmission routes were established, we calculated the transmission velocity by using the geographic distance and time between two sampling locations on the routes. We defined transmission velocity as follows:

(2) $$\;v_{i,j}=d_{i,j}/\left(t_{j}-t_{i}\right)\;$$

Here, $v_{i,j}$ is the transmission velocity (km/yr) along a route linked by samples *i* and *j*, $d_{i,j}$ is the distance between sampling locations where samples *i* and *j* were sampled, $t_{j}$ is the sampling time of sample *j*, and $t_{i}$ is the sampling time of sample *i*.

### Robustness of the NGDA model

To test whether the sample size would cause biases in the calculation of the transmission velocity by using NGDA, we performed random resampling of 25%, 50%, and 75% of all samples for each subtype of influenza virus 100 times (Fig. 4). The spread routes were then independently reconstructed using the resampled dataset, and the average spread velocity was calculated to make comparisons.

### Generalized additive models (GAMs)

We analyzed the association of several key environmental factors with the transmission velocity of IAV by using GAMs by referring to our previous study (Xu *et al*. 2019) implemented in R (R Core Team 2018) with the “mgcv” package. We used Pearson's correlation to calculate the correlation coefficients and the *t*‐test to test the difference in the transmit velocity in this study (Table 2).

### GAM model structure

The initial candidate models of transmission velocity included variables of human population density (Pop, person/km^2^), seasonal temperature (TempS, °C), and seasonal precipitation (PrecS, mm).

The initial model formulas are shown as follows:

(3) $$\begin{array}{ccc} \;V_{i,j} & = & \;a+b\left(\mathrm{Pop}\right)+c\left(\mathrm{PrecS}\right)\\ & & +d\left(\mathrm{TempS}\right)+e\left(\mathrm{LAT},\mathrm{LON}\right) \end{array}$$

Here, $V_{i,j}$ is the natural log‐transformed transmission velocity (plus 1 to avoid zero logarithm) of influenza virus from location *i* to *j*. *a* is the overall intercept; b(Pop) is smooth function of population density of location *j*; c(PrecS) is a smooth function of PrecS of location *j*; d(TempS) is a smooth function TempS of location *j*; and e(LAT,LON) is a smooth function used to control spatial autocorrelation. For details, see Table S1, Supporting Information.

### Model selection

We performed multi‐model inferences based on information theory (Burnham & Anderson 2002) to quantify the relative importance of predictors for influenza transmission velocity. We ranked the GAMs models based on AICc (Akaike's information criterion corrected for small sample sizes) and reported the models that were within 2 AICc units (ΔAICc ≤ 2) of the top model. We chose the model with the lowest AICc value among the reported models as the final model and excluded prediction variables that were not significant (*P* > 0.05) in the following analysis (Table S2, Supporting Information). All analyses were implemented in R using the *gam* function in the “mgcv” package and the *dredge* and *model.avg* functions in the “MuMIn” package.

## RESULTS

**Figure 2:**
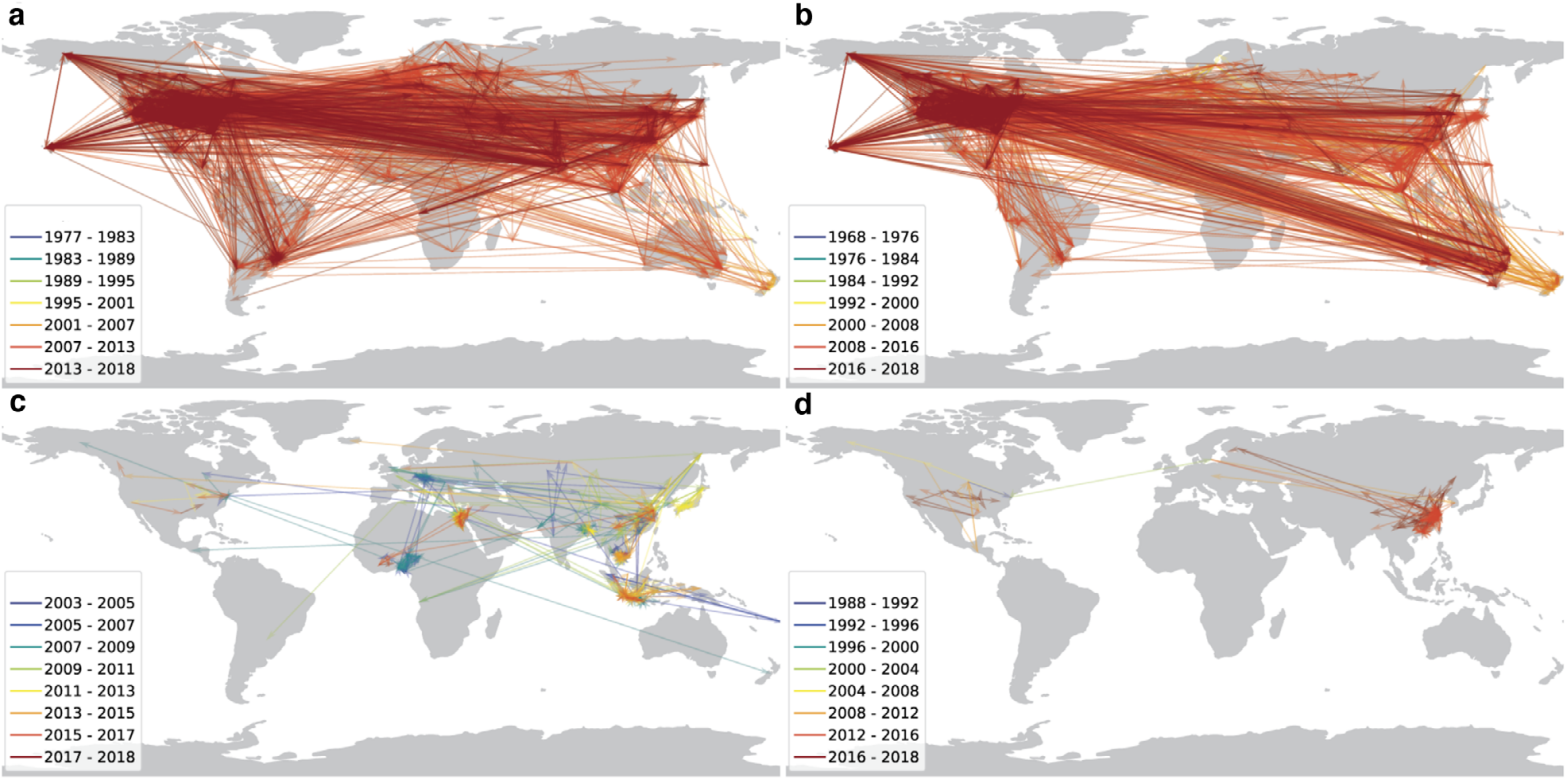
Global transmission patterns of (a) H1N1, (b) H3N2, (c) H5N1, and (d) H7N9 reconstructed based on the Nearest Genetic Distance Approach (NGDA). The lines indicate the reconstructed transmission routes of the IAVs. Each line represents a propagation event, and the arrow indicates the transmission direction. The color of the lines indicates the invasion time of the influenza virus.

NGDA analysis revealed that the transmission patterns of H1N1 and H3N2 were similar, being composed of highly inter‐regional long‐distance transmission routes across different continents (Fig. 2a,b). In contrast, the transmission patterns of H5N1 and H7N9 were composed of mainly localized transmission clusters and a number of inter‐regional long‐distance transmissions (Fig. 2c,d).

Based on the analysis of NGDA, we found that the estimated average transmission velocity of H1N1 (59 637 km/yr) was parallel to that of H3N2 (61 952 km/yr; *t* = −0.44, *P* = 0.66) but significantly larger than that of H5N1 (7672 km/yr; *t* = 14.5, *P* < 0.001) and H7N9 (11 682 km/yr; *t* = 12.5, *P* < 0.001). H1N1 and H3N2 had a higher proportion of human host (i.e. samples from humans) than H5N1 and H7N9. The proportion of human hosts was significantly correlated with the average velocity (*r* = 0.996, *P* = 0.004) and average distance (*r* = 0.993, *P* = 0.007) of the IAV (Table 1).

**Table 1:** The estimated average transmission velocity (km/yr) and distance (km) of influenza A viruses

Subtype	Average velocity (km/yr)	Average distance (km)	Number of transmission routes (*n*)	Proportion of human host
H1N1	59637 ± 3423	3152 ± 56	5086	0.77
H3N2	61952 ± 4085	3608 ± 65	4700	0.83
H5N1	7672 ± 1059	850 ± 55	851	0.09
H7N9	11682 ± 1765	982 ± 99	194	0.22

*n* is the number of samples when we calculated the average value and implemented *t*‐test between different subtypes. The value behind the “±” sign represents the standard error (SE) of average velocity and average distance. km/yr, kilometers per year.

**Figure 3:**
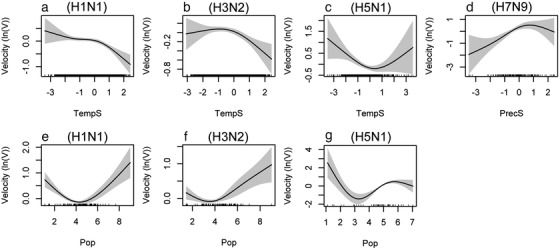
The significant associations of ln‐transformed transmission velocity of (a, e) H1N1, (b, f) H3N2, (c, g) H5N1, and (d) H7N9 with seasonal temperature (TempS), seasonal precipitation (PrecS), and population density (Pop). The solid line on the canvas indicates the fitted curve, and the gray interval indicates the 95% confidence interval. For definition of these variables, see Table S1, Supporting Information, and the main text.

Based on the results of our best selected models (Table S2, Supporting Information), variables showing significant associations with the transmission velocity of influenza are summarized in Fig. 3. The spread velocity of H1N1 and H3N2 showed a negative associations with the seasonal air temperature, a U‐typed association with population density (Fig. 3a,b,e,f). The spread velocity of H5N1 showed a U‐typed association with the seasonal air temperature and population density (Fig. 3c,g). The spread velocity of H7N9 showed a positive association with precipitation (Fig. 3d).

## DISCUSSION

By using NGDA, we reconstructed the global transmission patterns of 4 subtype of influenza A viruses. We found H1N1 and H3N2 showed faster, inter‐regional transmission, likely facilitated by human transportation via airplane; while H5N1 and H7N9 showed lower localized transmissions, likely facilitated by poultry transportation via railways or high ways. We found influenza viruses that were mainly carried by human spread faster in cold seasons, while H5N1, which were mainly carried by animals, spread faster in both warm and cold seasons. Some of the findings (e.g. seasonal climatic impacts) were consistent with those of previous results as revealed in laboratory or small‐scale studies, however, global analysis revealed the nonlinear impacts of environmental factors on transmission velocity of influenza viruses.

Our analysis showed that the proportion of human hosts was 77% for H1N1 and 83% for H3N2, supporting previous observations that human transportation was important in their spatial transmission (Krammer *et al*. 2018). Based on NGDA analysis, we found that H1N1 and H3N2 mainly displayed inter‐regional, long‐distance, and high‐speed transmissions, and their patterns were similar, likely facilitated by the similar aviation routes (Fig. 2a,b; Table 1). In contrast, H5N1 and H7N9 are known to be mainly transmitted by wildlife or domestic animals like chickens (Krammer *et al*. 2018) and the proportion of animal hosts was 91% and 79%, respectively, supporting the previous observations (Balcan *et al*. 2009; Chattopadhyay *et al*. 2018; Pei *et al*. 2018). The global transmission patterns of H5N1 and H7N9 were mainly composed of clusters with local short‐distance transmission, likely driven by local or regional poultry transportation as suggested before (Fournie *et al*. 2013) (Fig. 2c,d; Table 1), or by resident birds. However, they also displayed a number of long‐distance transmission routes, likely driven by migratory birds or international transportation of poultry products (Olsen *et al*. 2006).

In this study, we quantified associations of transmission velocity of influenza virus with the seasonal temperature and precipitation. We found that H1N1 and H3N2 spread faster in cold seasons (Table 2; Fig. 3a,b), which is consistent with our knowledge and the results of previous studies (Lowen *et al*. 2007; Tamerius *et al*. 2013; Huang *et al*. 2016; Gomez‐Barroso *et al*. 2017). However, we found that H5N1 spread faster in both cold and warm seasons (Table 2; Fig. 3c); the nonlinearity might be caused by migrations of wild birds which travel back and forth between cold and warm seasons for breeding and foraging (Olsen *et al*. 2006). We found H7N9 spread faster in wet seasons (Table 2; Fig. 3d), which is contradicting with some observations that influenza virus favors dry conditions (Lowen *et al*. 2007; Tamerius *et al*. 2013; Huang *et al*. 2016; Gomez‐Barroso *et al*. 2017).

**Table 2:** Significant associations of the transmission velocity of influenza A virus with environmental factors

Predictor variable	H1N1*P*‐value	H3N2*P*‐value	H5N1*P*‐value	H7N9*P*‐value
PrecS	None	None	None	< 0.01(+)
TempS	< 0.01(‐)	< 0.01(‐)	< 0.01(U)	None
Pop	< 0.01(U)	< 0.01(U)	< 0.01(U)	None
LAT, LON	None	< 0.01	< 0.01	None

“U” represents overall U‐shaped association between transmission velocity and variables within the range of most samples. Significance level was set as: *P* < 0.05. Pop, population density; TempS, seasonal temperature; PrecS, seasonal precipitation; LAT, LON, spatial autocorrelation; none, no significant associations.

We analyzed associations of influenza transmissions with human population density. We found that human population density had a U‐shaped association with H1N1, H3N2, and H5N1, indicating that they spread faster in regions with both high and low human activities (Table 2; Fig. 3e–g). It is reasonable that influenza virus spread faster in high‐density places due to frequent contact of people facilitated by modern transportation (Gao 2014; Dalziel *et al*. 2018). The plausible explanation to the high spread velocity of influenza virus in sparsely dense places was that the countries with low population density like Australia, Mongolia, and Canada heavily depended on air transportation, which might facilitate the spread of the influenza virus. This observation needs further investigation in future studies.

The direct estimation of transmission velocity of pathogen is important but still rare in literature. The average transmission velocity of rodent‐borne plague was estimated to be 40.1 km/yr in China from 1772 to 1964 (third pandemic) (Xu *et al*. 2014), 45–87 km/yr in USA from 1900 to 1966 (third pandemic) (Adjemian *et al*. 2007), and 341.9–643.7 km/yr from 1347–1350 in Europe (Black Death) (Noble 1974). In this study, the transmission velocity of H1N1 (59 637 km/yr), H3N2 (61 952 km/yr), H5N1 (7672 km/yr), and H7N9 (11 682 km/yr) was estimated to be much higher than that of plague. This is because plague was mainly transmitted by animal hosts or less advanced human transportation systems in ancient time, while the current transmission of influenza was facilitated by modern fast transportation tools, such as airplanes and railways. The higher transmission velocity of influenza virus indicated that airplane and railway or high‐way transportation were the major driving forces of geographic transmissions of influenza virus. Unfortunately, we did not have inter‐location route data of airplane, railways, or high way, and were not able to qualify their associations with the transmission velocity of influenza viruses.

**Figure 4:**
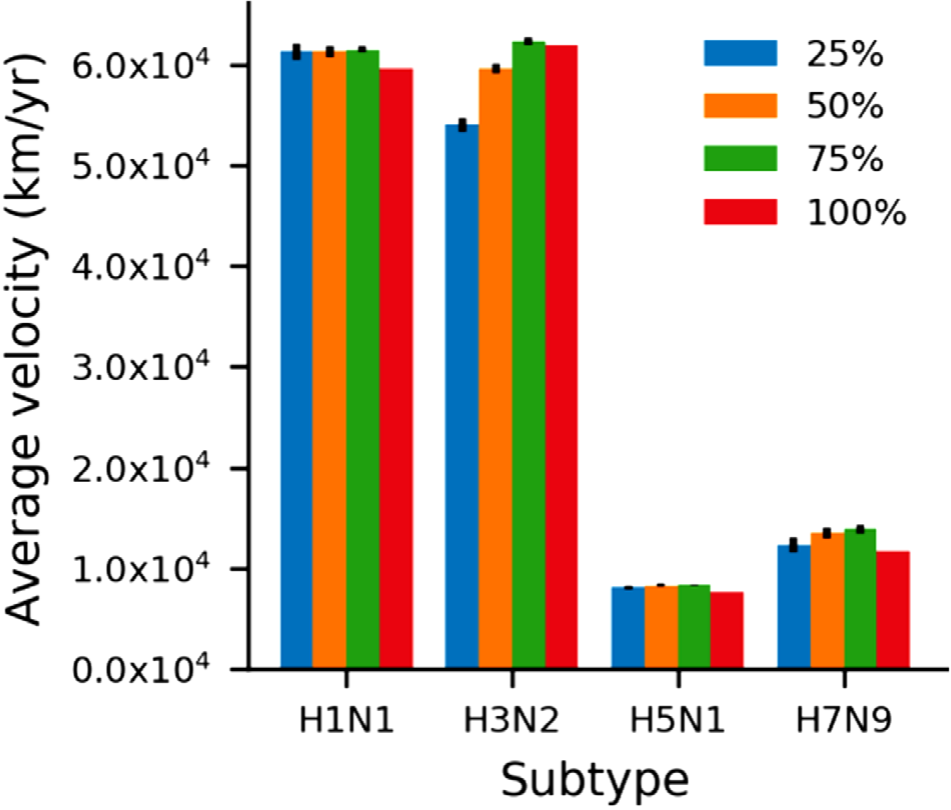
Comparisons of estimated average velocity of H1N1, H3N2, H5N1, and H7N9 using resampling data of 25%, 50%, 75%, and 100% of all data. This analysis indicates that the NGDA was robust to missing samples in calculating the transmission velocity.

Our NGDA method has several advantages in reconstructing transmission patterns of influenza virus. The genomic data of influenza virus accumulated quickly in the public database, which impose a challenge of calculation efficiency using traditional modeling approaches. Missing data are common in disease sampling which may cause serious biased estimations. Using a resample approach with NGDA method, we found the results of resampled data of 25%, 50%, and 75% were similar to that of the whole data (Fig. 4), indicating NGDA is robust to missing data in calculating transmission velocity of influenza viruses. However, it should be pointed out that the transmission route was mapped using NGDA based on its genetically closest relatives, which were not necessary the real ancestors but “proxy ancestors” of transmission. Thus, as shown in Fig. 1, missing data would cause errors in reconstruction of real transmission routes of some samples. We should be very cautious in explaining the sources and routes of disease transmission in the circumstance of missing data in both time and space.

Considering the huge amount of damage caused by influenza, it is necessary to develop effective strategies for prevention and control of IAVs. Our results provide some cues for managing influenza transmission. For viruses mainly carried by human‐like H1N1 and H3N2, we should take quarantine measures for passengers at the airports, particularly for those located within the transmission hotpots, to prevent cross‐wired long‐distance transmission. Due to highly frequent global transmission of H1N1 and H3N2 across different regions of the world, it is necessary to develop polyvalent vaccines for preventing the influenza pandemic based on monitoring information of variants of influenza virus from the source regions. For viruses mainly carried by animals like H5N1 and H7N9, prevention measures such as monitoring poultry transportation along railways and high‐ways and closing live poultry market in pandemic period should be taken, so as to prevent localized transmissions. For control of H5N1, careful monitoring of water habitats along the major pass ways of migratory birds are necessary to reduce contact with domestic animals or people, particularly in autumn.

## CONFLICT OF INTEREST

The authors declare no conflict of interest.

## AUTHOR CONTRIBUTIONS

Conceptualization: Z.Z. and L.X.; data curation: C.C. and J.L.; formal analysis: C.C., Z.Z., and L.X.; investigation: Z.Z.; methodology: Z.Z, C.C., and L.X. Software: C.C.; visualization: C.C and J.L.; writing, original draft: C.C., J.L., and Z.Z.; writing, review, editing: C.C., J.L., W.L., L.X., and Z.Z.

## Supporting information


**Table S1** Data information about the predictor variables.
**Table S2** Multi‐model inference results.
**Figure S1** Geographical distribution of samples of H1N1 (A), H3N2 (B), H5N1 (C), and H7N9 (D) in this study. Blue dots represent the sampling locations of influenza virus.
**Figure S2** Temporal frequency of samples of H1N1 (A), H3N2 (B), H5N1 (C), and H7N9 (D) in this study.
