# Supplementary material for: Modeling analysis revealed the distinct global transmission patterns of influenza A viruses and their influencing factors
Source: Integr Zool. 2020 Aug 6;16(6):788–97. doi: 10.1111/1749-4877.12469 (PMC9292709; doi:10.1111/1749-4877.12469)
Supplement: Supplementary file 1 — Table S1 Data information about the predictor variables. Table S2 Multi‐model inference results. Figure S1 Geographical distribution of samples of H1N1 (A), H3N2 (B), H5N1 (C), and H7N9 (D) in this study. Blue dots represent the sampling locations of influenza virus. Figure S2 Temporal frequency of samples of H1N1 (A), H3N2 (B), H5N1 (C), and H7N9 (D) in this study. [file INZ2-16-788-s001.docx]

**SUPPLEMENTARY MATERIALS**

**Table S1** Data information about the predictor variables. MT represent monthly average daily temperature, MP represent monthly precipitation, YT represent annual average temperature and AP represent annual precipitation.

| Variables | Description | Data Type | Source |
| --- | --- | --- | --- |
| TempS | MT - YT | raster | http://www.cru.uea.ac.uk/data |
| PrecS | MP - AP/12 | raster | http://www.cru.uea.ac.uk/data |
| Pop | Human population density of 2018 | country-level | http://worldpopulationreview.com |

**Table S2** Multi-model inference results. We only reported the top models sorted by AICc value with Delta AICc less than 2. “Y” indicated that this variable was included in this model and “N” indicates this variable was excluded in this model due to multi-model inference process. “*****” indicated this variable was also significant (p < 0.05) in GAM and included in the final model. For definition of these variables, see Table S1 and the main text.

| **Serotype** | **Delta AICc** | **PrecS** | **TempS** | **Pop** | **LAT_LON** |
| --- | --- | --- | --- | --- | --- |
| **H1N1** | 0 | N | Y* | Y* | Y* |
| **H1N1** | 1.33 | Y | Y | Y | N |
| **H3N2** | 0 | N | Y* | Y* | Y* |
| **H3N2** | 1.2 | Y | Y | Y | Y |
| **H5N1** | 0 | Y | Y* | Y* | Y* |
| **H5N1** | 0.37 | N | Y | Y | Y |
| **H7N9** | 0 | Y* | N | Y | N |

**
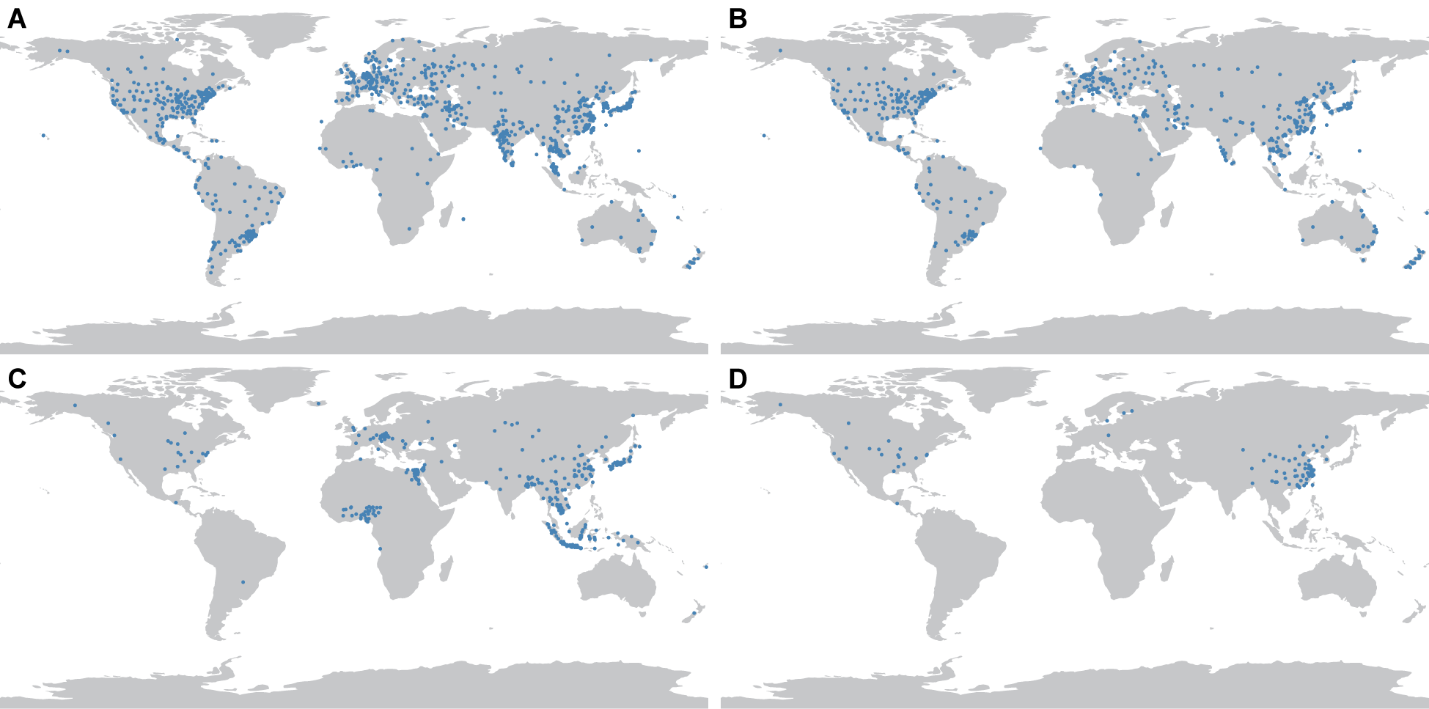
**

**Figure S1** Geographical distribution of samples of H1N1 (A), H3N2 (B), H5N1 (C), and H7N9 (D) in this study. Blue dots represent the sampling locations of influenza virus.


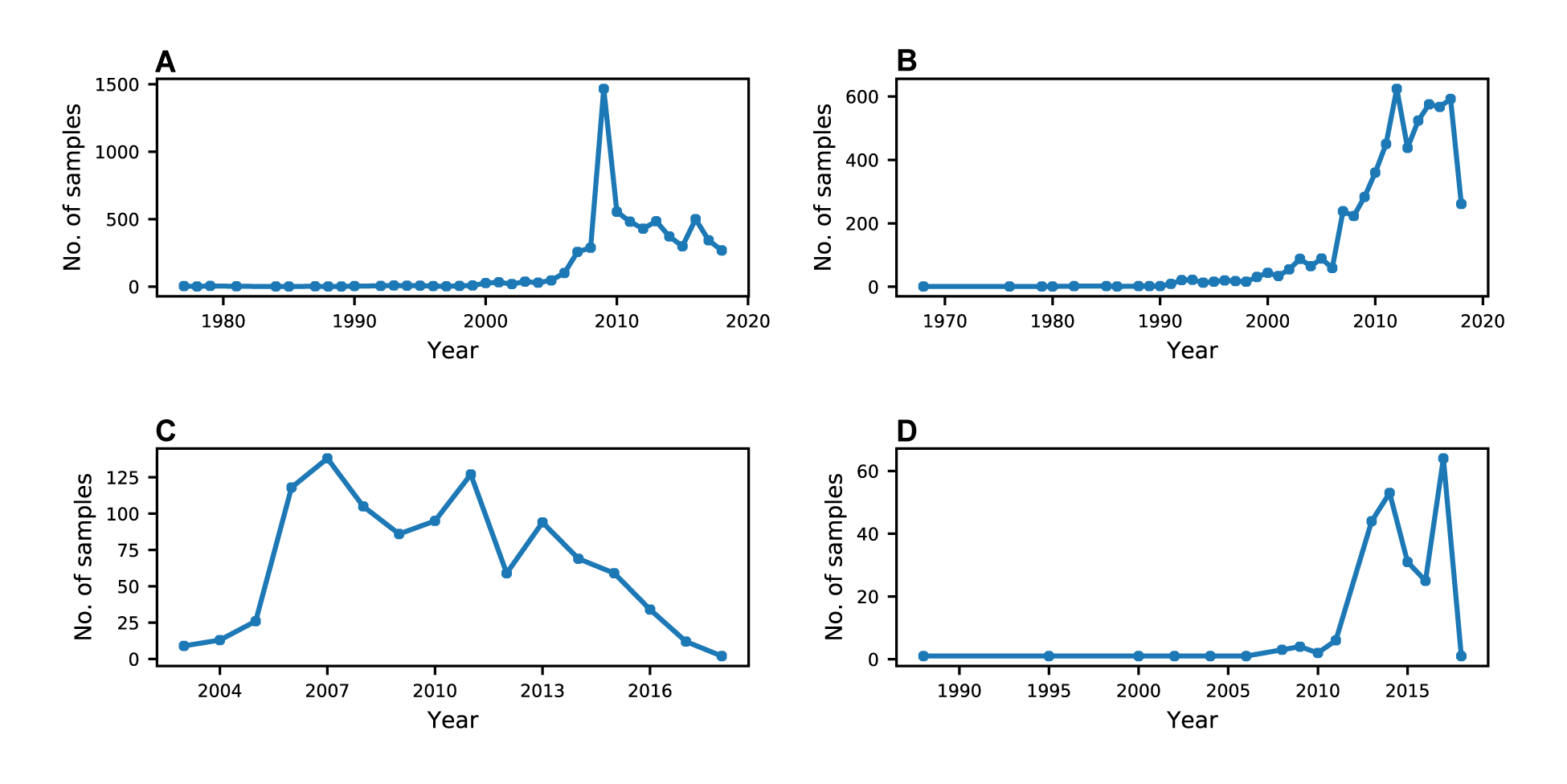


**Figure S2** Temporal frequency of samples of H1N1 (A), H3N2 (B), H5N1 (C), and H7N9 (D) in this study.
